# Tricuspid valve repair in isolated tricuspid pathology: a 12-year single center experience

**DOI:** 10.1186/s13019-020-01369-8

**Published:** 2020-11-16

**Authors:** Alina Zubarevich, Marcin Szczechowicz, Andreas Brcic, Anja Osswald, Konstantinos Tsagakis, Daniel Wendt, Bastian Schmack, Michel Pompeu B. O. Sá, Jef Van den Eynde, Arjang Ruhparwar, Konstantin Zhigalov

**Affiliations:** 1 Department of Thoracic and Cardiovascular Surgery, West German Heart and Vascular Center, University of Duisburg-Essen, Essen, Germany; 2 Department of Cardiovascular Surgery Essen-Huttrop, University Hospital Essen, Essen, Germany; 3 Department of Anesthesiology, University Hospital Essen, Essen, Germany; 4 Department of Cardiovascular Surgery at the Pronto Socorro Cardiológico de Pernambuco (PROCAPE), Recife, PE Brazil; 5 Department of Cardiovascular Diseases, University Hospitals Leuven, Leuven, Belgium

**Keywords:** Tricuspid valve, Tricuspid valve regurgitation, Isolated tricuspid valve pathology

## Abstract

**Objectives:**

Long-term data on isolated surgical tricuspid valve procedures is limited. Current guidelines on heart valve disease recommend valve repair over valve replacement. In this study we report our 12-year single-center experience with isolated surgical tricuspid valve repair in patients with various tricuspid valve pathologies.

**Methods:**

Between May 2007 and December 2019, 26 consecutive patients underwent isolated tricuspid valve annuloplasty/repair for various indications. In 18 patients (69.2%) an open ring or band annuloplasty (26.9 and 42.3%, respectively) was performed, 5 patients (19.2%) underwent a tightening of the annulus using the DeVega technique, 5 patients (19.2%) had a leaflet reconstruction with patch or bicuspidalization and in 3 patients (11.5%) a leaflet debridement was performed. In 15.4% of the cohort a combination of the techniques was utilized.

**Results:**

The mean follow-up time was 2.1 (0.3–5.0) years. Early survival at 30 days after surgery was 84.6%. Mean hospital stay was 11 (6.7–16) days. One-year survival was 73%. No patient required a redo procedure on the tricuspid valve during follow-up.

**Conclusion:**

Tricuspid valve repair is suggested as a treatment of choice according to recent guidelines on heart valve disease. If chosen correctly, various repair techniques provide good long-term results. Tricuspid valve repair may be safely applied in patients undergoing surgical isolated tricuspid valve procedures.

## Introduction

Tricuspid regurgitation (TR) often has a secondary nature, resulting from volume or pressure overload in the presence of right ventricular (RV) failure and annular dilatation with structurally normal leaflets [1]. The most common causes of primary TR are infective endocarditis, mechanical damage by pacemaker wires, rheumatic heart disease, Ebstein anomaly, and drug abuse-induced tricuspid valve endocarditis. Indication and timing of surgical intervention remains controversial due to the limited availability of data on isolated tricuspid valve procedures.

Current guidelines suggest that surgery should be carried out as early as onset of signs of RV dysfunction [2]. Valve repair should be preferred over valve replacement for secondary TR, based on the surgeon’s experience, specifics of the valve pathology, and the patient’s condition. Valve replacement should be restricted to those pathologies with severely destroyed and tethered leaflets and to cases with a severe annular dilatation [2]. Most of the current data on surgical therapy of severe TR originate from concomitant procedures on the left sided valves [3, 4]. Though isolated surgical procedures on the tricuspid valve are less common, some reports have compared isolated surgical tricuspid valve repair and replacement and demonstrated that both are feasible for the patients with isolated TR [5].

The aim of this study is to present our 12-years single center experience with isolated surgical tricuspid valve replacement in patients with primary and secondary TR.

## Material and methods

### Study population

Between May 2007 and December 2019, 26 consecutive patients underwent isolated tricuspid valve annuloplasty/repair (TAP) at our institution because of various indications. The surgical indications were made following the current guidelines [2].

### Study design

The study is a retrospective review of prospectively collected data. The data collected as a part of the institutional database included detailed information on patients’ demographics, baseline clinical characteristics and their laboratory, echocardiographic and hemodynamic parameters, as well as intraoperative variables and postoperative outcomes. The study was approved by the local ethics committee.

### Study endpoints

The primary endpoint was death in short- and mid-term follow-up. Secondary endpoints were adverse events and other postoperative characteristics during the follow-up period.

Additionally, the cohort was divided in two groups: in group 1 (*n* = 8) patients underwent a TAP on the arrested heart while the patients in group 2 (*n* = 18) underwent a beating heart procedure. Survival was compared between these two groups.

### Surgical techniques

In most patients the procedure was performed via median sternotomy except for 4 patients in whom a right anterolateral thoracotomy was used. All procedures were performed on cardiopulmonary bypass (CPB) with a standard bicaval cannulation technique of ascending aorta, vena cava superior and inferior. Eight (30.8%) patients were operated under cardioplegic arrest while the other 18 (69.2%) received a beating heart procedure. Cannulation of the groin vessels was used in those patients with right anterolateral access. Various valve repair techniques were used according to the pathology. In 4 (15,4%) of the cases a combination of techniques was used to achieve a competent tricuspid valve morphology. The chest was closed in a regular manner using steel wires.

### Anticoagulation protocol

At ≥12 h after the procedure, when the chest tube drainage decreased to ≤50 mL per hour and the coagulation profile returned to normal or near-normal levels, intravenous heparin infusion was commenced to maintain an activated partial thromboplastin time (aPTT) between 50 and 70 s. After removal of the chest tubes and starting oral medication, phenprocoumon was administered to maintain an international normalized ratio (INR) between 2.5 and 3.5. Heparin infusion was continued until the INR target range was attained. Three months after the surgery the patients discontinued phenprocoumon therapy, if no other reason for anticoagulation existed.

### Statistical analysis

The data were analyzed using IBM SPSS version 26 (IBM Corp., Chicago, Ill., USA) and R software v.3.4.3 (R Foundation for Statistical Computing, Vienna, Austria). The data were checked for normality using the Shapiro-Wilk test. As data were not normally distributed, continuous variables are expressed as the medians (interquartile range, IQR) and were compared between groups using the Mann-Whitney U test. Categorical variables are expressed as frequencies and percentages and were compared between groups using the Chi-squared test. We used the Kaplan-Meier method to analyze survival. The significance of survival differences between the groups was assessed with Log-Rank and Breslow tests. A value of *p* < 0.05 was considered to be statistically significant.

### Follow-up

The follow-up was performed either by telephone interview with patients’ GPs or by telephone contact with patients and/or family members. The data on mortality was provided by the local city hall’ bureau of vital statistics if the information was unobtainable from the medical records.

## Results

### Baseline characteristics

Preoperative demographic and clinical characteristics of the entire cohort of 26 patients (61.5% females, *n* = 16) are presented in Table 1. Twenty-one (80.8%) patients presented with severe TR, 42.3% (*n* = 11) suffered from pulmonary hypertension, and 26.9% (*n* = 7) presented with at least mild impairment of the right ventricular function as determined by transthoracic or transesophageal echocardiography. Twelve (46.2%) patients had an infective endocarditis and received antibiotics at admission. Seven (26.9%) patients were active intravenous drug abusers. Seven (26.9%) patients had a history of previous sternotomy. Also, 7 (26.9%) patients suffered from cardiac decompensation prior to surgery and 2 (7.7%) of them were mechanically ventilated upon referral.

**Table 1:** Baseline characteristics

Characteristics	Value
**Demographic data**	
Age (years)	58.1 ± 21.6
Female	16 (61.5%)
BMI	24.7 ± 4.7
**Angina pectoris**	
CCS III	3 (11,5%)
**Dyspnea**	
NYHA I-II	6 (23%)
NYHA III-IV	20 (76.9%)
Pulmonary edema	3 (11.5%)
Peripheral edema	16 (61.5%)
Chronic kidney injury (GFR 30–60)	19 (73.1%)
Coronary artery disease	10 (38.5%)
Previous PCI	5 (19.2%)
Mitral regurgitation ≥II	5 (19.2%)
Septic embolization	3 (11.5%)
Previous decompensation	7 (26.9%)
Previous cardiopulmonary resuscitation	1 (3.8%)
Mechanical ventilation	2 (7.7%)
**Mechanical circulatory support**	
ECLS	1 (3.8%)
**Cardiovascular risk factors**	
Hyperlipidemia	12 (46.2%)
Hypertension	18 (69.1%)
Smoking history	13 (50%)
**Diabetes mellitus**	
NIDDM	3 (11.5%)
Pulmonary hypertension	11 (42.3%)
**Atrial fibrillation**	15 (57.7%)
Paroxysmal	6 (23.1%)
Permanent	9 (34.6%)
Previous stroke	4 (15.4%)
History of sternotomy	7 (26.9%)
**Prior cardiac surgery**	
Coronary surgery	2 (7.7%)
Aortic valve surgery	2 (7.7%)
Mitral valve surgery	4 (15.4%)
Tricuspid valve surgery	3 (11.5%)
Pacemaker implantation	6 (23.1%)
Infection	12 (46.2%)
Antibiotics	12 (46.2%)
Intravenous drugs abuse	7 (26.9%)
Left ventricular ejection fraction	median 60 (54,5–60)
EuroSCORE II	median 4.9 (3.4–12.0)
**Right ventricular function imparement**	
Mild (TAPSE< 15)	5 (19.2%)
Moderate (TAPSE < 10)	2 (7.7%)
Right ventricular-dilatation (mid-cavity-diameter > 4 cm)	11 (42.3%)
Pulmonary hypertension (mean > 40 mmHg)	11 (42.3%)
Severe tricuspid regurgitation (≥III°)	21 (80.8%)

### Intraoperative data

Eleven (42.3%) patients underwent urgent surgery and in 7.7% (*n* = 2) of the cases an emergent procedure had to be performed due to hemodynamic instability. According to the pathology of the TR, various surgical techniques were applied to achieve a satisfactory valve competence (Table 2). In 4 (15.4%) cases a combination of the described techniques was performed. Four (15.4%) patients underwent an endoscopic procedure via anterolateral thoracotomy, in 2 of whom an access conversion to median sternotomy had to be performed due to severe pleural and pericardial adhesions. In 8 (30.8%) patients the procedure was performed in cardioplegic arrest with crystalloid cardioplegia and the other 18 (69.2%) underwent a beating heart procedure. Median CPB-time was 62 (45.7–79.7) min (Table 2).

**Table 2:** Intraoperative characteristics

Characteristics	Value
**Tricuspid valve-pathology**	
Non-rheumatic tricuspid regurgitation	10 (38.5%)
Infective endocarditis	8 (30.8%)
Pacer leads-associated pathology	5 (19.2%)
Hedinger Syndrome	1 (3.8%)
Morbus Ebstein	1 (3.8%)
Port-infection	1 (3.8%)
**Urgency of procedure**	
elective	13 (50%)
urgent	11 (42.3%)
emergent	2 (7.7%)
**Type of tricuspid valve repair**	
Open ring	7 (26.9%)
Cosgrove band	11 (42.3%)
DeVega -procedure	5 (19.2%)
Leaflet reconstruction	6 (23.1%)
Leaflet debridement	3 (11.5%)
Isolated procedure	17 (65.4%)
**Concomitant procedures**	
Epimyocardial leads	1 (3.8%)
Persistent foramen ovale closure	3 (11.5%)
LAA-Ligation	1 (3.8%)
Pacer-leads-extraxtion	2 (7.7%)
Port-extraction	1 (3.8%)
**Surgical access**	
Right anterolateral thoracotomy	4 (15.4%)
Median sternotomy	22 (84.6%)
Beating heart	18 (69.2%)
Plegia	8 (30.8%)
CPB time in min	62 (45.7–79.7)

### Postoperative data

**Fig. 1:**
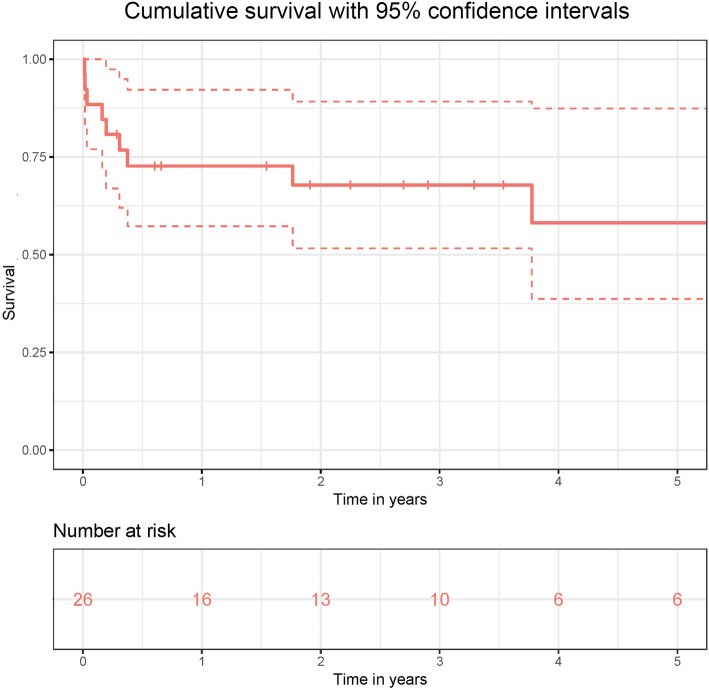
Cumulative survival with 95% confidence intervals

Postoperative adverse events are listed in the Table 3. The median follow-up time was 2.1 (0.3–5.0) years. The 30-day survival was 84.6%. Median hospital stay was 11 (6.7–16) days (Fig. 1). After 12 months, 73% of the patients were alive (Table 3). There was no significant difference in survival between those patients undergoing TAP on an arrested versus on a beating heart (*p* = 0.4 for short-term survival and 0.6 for mid-term survival).

**Table 3:** Postoperative characteristics

Characteristics	Value
Atrial fibrillation	3 (11.5%)
Venticular extrasystole	2 (7.7%)
Low output syndrome	1 (3.8%)
MCS	1 (3.8%)
**Renal failure**	15 (57.6%)
Conservative treatment	10 (38.5%)
Hemofiltration	5 (19.2%)
**Respiratory insufficiency**	
Forced respiratpry therapy	14 (53.8%)
Re-Intubation	4 (15.4%)
**Pleural effusion**	19 (73.1%)
Conservative treatment	14 (53.8%)
Drainage	3 (11.5%)
Pacemaker-implantation	1 (3.8%)
**Cerebrovasular accident**	2 (7.7%)
Stroke	1 (3.8%)
Intracerebral bleeding	1 (3.8%)
**Re-intervention on the tricuspid valve**	0
Follow-up, years	median 2.07 (0.3–5)
Hospital stay, days	median 11 (6.7–16)
Death during follow-up	10 (38.5%)
In-hospital death	4 (15.4%)

## Discussion

Isolated surgical procedures on the tricuspid valve remain a controversial topic, as this type of surgery is known to be associated with high morbidity and mortality [5]. Data on the topic are rather limited [6]. Only 20% of all the surgeries involving the tricuspid valve are isolated procedures [7], and they should be performed rather sooner than later. The mortality of the isolated procedure is higher than of an isolated procedure on any other valve or a concomitant procedure involving tricuspid valve. Part of this high mortality might be explained due to underlying lung pathologies causing the TR and which may impair postoperative recovery. The surgical risk increases with increasing severity of right and left ventricular functional impairment and progression of right ventricular dilatation [8]. In our cohort, patients who were in a critical condition – i.e., those who were ventilated prior to surgery, those who suffered from severe pulmonary hypertension after a prior mitral valve procedure [9], or those in acute septic shock due to acute infective endocarditis - had the worst outcomes. Given the limited sample size, we were unable to run a regression model to study these factors as being potential predictors of mortality. However, it is likely that these may have had a negative impact on postoperative survival of the overall cohort. Indeed, pulmonary hypertension is known to be a negative predictive factor for operative mortality and is seen in many patients who suffer from secondary TR [10, 11].

Previous studies have shown high early- and long-term TR recurrence rates after tricuspid valve repair despite the use of the annuloplasty rings [12]. In our cohort we did not observe any cases of recurrent TR during follow-up, neither after ring-annuloplasty nor after any other surgical technique. McCarthy et al. [12] emphasized an elevated risk of TR recurrence of up to 20% after 8 years in patients undergoing DeVega procedure compared to the patients receiving annuloplasty rings or Cosgrove bands and thus suggested to abandon this technique for the secondary TR. Another group from the University of Rochester reported about 24% of TR recurrences after 5 years in patients with functional TR [13]. In our study, patients who were treated with DeVega technique did not need any repeated intervention within the follow-up. However, in these cases, the indication for the procedure was not functional TR, but either mechanical damage caused by pacemaker wires or an infective vegetation in the absence of leaflet destruction and right ventricular dilatation. Hence, the DeVega procedure was performed mainly to stabilize the TV annulus from the further dilatation, which was in these cases unlikely to happen. In agreement with the results from our study, Eichhorn at al [14]. showed satisfactory results after DeVega annuloplasty in patients with primary TR. This might lead us to argue that this method should not be entirely abandoned, but rather be considered for specific indications in carefully selected patients. Additionally, McCarthy at al [12]. suggested to replace the transvenous pacer leads with the epimyocardial leads, which was performed in all patients in our cohort who required a pacemaker prior to the tricuspid valve procedure.

Patients who received ring or band annuloplasty were not re-operated on the tricuspid valve during the follow-up time (Table 3). Recurrence of TR might be an important reason for the diminished mid- and long-term postoperative survival observed in other reports about patients after tricuspid valve procedures, as redo procedures on the tricuspid valve are associated with extremely high hospital mortality rates up to 37%. In these cases, the redo surgery is mostly performed despite impaired right ventricular function, progressing pulmonary hypertension, and right ventricular dilatation [15, 16].

Two other patients underwent an urgent procedure due to acute endocarditis with impaired coagulation due to sepsis, which is known to increase the risk for bleeding [17].

The overall results of this study showed a 30-day survival of 84.6% and 1-year survival of almost 73%. These results differ slightly from those of Moraca et al. [18], who reported a 1-year survival rate of 80%. Our results differ also from the recent data of the meta-analysis from the Cleveland Clinic, which reported a pooled operative mortality of 8.4% and a late mortality of 12.7% [5]. In our study, the four patients who died within 30 days postoperatively were all in a critical condition before the surgery. Two of them were elderly patients undergoing an urgent redo procedure with manifest pulmonary hypertension (mean pulmonary artery pressure, mPAP> 40 mmHg) and logistic EuroSCORE > 30%. Another patient presented with acute infective endocarditis and severe septic shock who received a peripheral extracorporeal life support (ECLS) preoperatively, a combination which is known to be associated with high mortality rates [19]. The fourth patient died from a cerebrovascular bleeding. We found no significant difference in survival between patients operated on an arrested or beating heart, suggesting that both procedures may be equally safe.

There are only few studies reporting about isolated tricuspid valve procedures. Hence, any new data on this matter are of great interest. Unfortunately, the limited cohort size did not allow to study subgroups according to the tricuspid valve pathology. This could however have provided some additional information on the outcomes for every specific pathology. Furthermore, this was a retrospective observational study and might therefore be susceptible to confounding.

## Conclusions

Analyzing a 12-year single center experience with isolate tricuspid valve disease, this study demonstrated that tricuspid valve repair can be performed in these patients with acceptable postoperative morbidity and mortality. Even though redo procedures on the tricuspid valve carry a high mortality, it might be lowered by performing the surgery as soon as the indication occurs. Recurrent TR has a considerably negative impact on the postoperative survival. Thus, the surgical technique should be chosen carefully, according to the patient’s valve pathology.

## Data Availability

The datasets used and analysed during the current study are available from the corresponding author on reasonable requests.
